# Nutritional Compositions, Phenolic Contents, and Antioxidant Potentials of Ten Original Lineage Beans in Thailand

**DOI:** 10.3390/foods11142062

**Published:** 2022-07-12

**Authors:** Yuraporn Sahasakul, Amornrat Aursalung, Sirinapa Thangsiri, Pitthaya Wongchang, Parichart Sangkasa-ad, Aphinya Wongpia, Auytin Polpanit, Woorawee Inthachat, Piya Temviriyanukul, Uthaiwan Suttisansanee

**Affiliations:** 1 Food and Nutrition Academic and Research Cluster, Institute of Nutrition, Mahidol University, Salaya, Phuttamonthon, Nakhon Pathom 73170, Thailand; yuraporn.sah@mahidol.ac.th (Y.S.); amornrat.aur@mahidol.ac.th (A.A.); sirinapa.tha@mahidol.ac.th (S.T.); woorawee.int@mahidol.ac.th (W.I.); piya.tem@mahidol.ac.th (P.T.); 2 Biotechnology Research and Development Office, Department of Agriculture Rangsit-Nakorn Nayok, Rangsit (Klong 6), Thanyaburi, Pathum Thani 12100, Thailand; pitthaya@hotmail.com (P.W.); psk50_2003@hotmail.com (P.S.-a.); aphinya.wongpia@gmail.com (A.W.); 3 Chiang Mai Field Crops Research Center, Department of Agriculture, Nong Han, San Sai District, Chiang Mai 50290, Thailand; auytin1804@hotmail.com

**Keywords:** antioxidant activities, bioactive compounds, proximate, *Phaseolus lunatus*, *Phaseolus vulgaris*, *Vigna umbellata*, *Vigna angularis*, *Vigna mungo*, *Vigna radiata*, *Glycine max*

## Abstract

Legumes and pulses are nutrient-dense foods providing a good source of protein, complex carbohydrates, fiber, vitamins, minerals, and bioactive compounds. To breed a new lineage of beans with specific nutritional and health beneficial purposes, more information on original lineage beans must be obtained. However, data concerning the nutritive compositions, total phenolic contents (TPCs), and health benefits regarding the antioxidant potentials of some original lineage beans in Thailand remain scarce, causing difficulty in decisional selection to breed a new lineage. Thus, this study aimed to examine the nutritional values (proximate compositions, vitamins, and minerals), TPCs, and antioxidant activities of ten original lineage bean cultivars in *Glycine*, *Phaseolus,* and *Vigna* genera from Genebank, Department of Agriculture (DOA), Thailand. The results indicated that beans in the *Glycine* genus potentially provided higher energy, protein, fat, and calcium contents than other genera, while the *Phaseolus* genus tended to provide higher carbohydrate and dietary fiber. Specifically, lima bean cultivar ‘38’ exhibited high vitamin B1, and red kidney bean cultivar ‘112’ exhibited high potassium content. Beans in the *Vigna* genus exhibited high TPCs and antioxidant activities. However, their nutritional compositions were markedly varied. The results of this work could support bean consumption as a feasible alternative diet and be used as a reference for future bean breeding (within the same genera) of a new lineage with particular nutritional requirements and health potentials.

## 1. Introduction

Malnutrition is a condition that occurs when one’s diet is unbalanced with under-nutrition, over-nutrition, or both, all of which could coexist and result in adverse health effects [1,2]. In 2020, the World Health Organization (WHO) reported that 149.2 million children under five years of age were stunted, 45.4 million were wasted, and 38.9 million children were overweight [1,2]. Interestingly, 45% of deaths in children under five years old were associated with under-nutrition, indicating that malnutrition is a severe global burden, especially for infants, children, and adolescents in low-income countries [1]. In addition, more than 12 million adult deaths were due to poor diet and diet-related diseases [3]. The primary dietary risks include low consumption of fruits, vegetables, legumes, nuts, seeds, and whole grains and high intake of red meat, processed meat, and sweetened beverages [3]. However, there has been little progress in tackling these global issues [4]. One of the approaches that might help this adverse situation is to provide high-quality food products at accessible prices. Plant-based diets have gained attention, as measured by the rapid expansion of the plant-based food sector [5]. This could be due to several plant ingredients that have been reported to be rich in macro- and micronutrients with health-promoting potential and sustainability [6]. Several plants are used for a plant-based diet, including sunflower, peas, rice, wheat, lentils, fava beans, and soybeans [6].

Beans are a crucial food ingredient in various diets throughout the world, making them one of the world’s most important legumes. Legumes belong to the Leguminosae family, which possesses 600 genera and over 13,000 species [7]. Data in 2011–2013 indicated that legumes were consumed the most in Latin America and the Caribbean (about 33–34 g per capita per day), followed by sub-Saharan Africa and South Asia, while those in Southeast Asia, including Thailand, consumed approximately 10 g of legumes per capita per day [8]. Beans have been recognized as food ingredients rich in protein, vitamins, minerals, and phytonutrients [9]. For example, the common bean (*Phaseolus vulgaris*) variety ‘American black’ cultivated in Mexico possesses protein, total dietary fiber, and lipids at 20.4, 23.4, and 3.6 g/100 g of dry sample, respectively. In contrast, the *Phaseolus coccineus* (scarlet runner bean) variety ‘Purple scarlet runner’ showed protein, total dietary fiber, and lipids at 18.0, 21.8, and 2.8 g/100 g of dry sample, respectively [10]. Interestingly, four varieties of soybean (*Glycine max*) grown in Brazil, namely ‘Conquista’, ‘BRSMG 800A’, ‘BRSMG 790A’, and ‘MGBR07-7141’, contained protein content ranging from 35.35 to 39.80 g/100 g dry sample, lipid ranging from 18.15 to 19.50 g/100 g dry sample, and crude fiber ranging from 6.70 to 10.70 g/100 g dry sample [11]. Such amounts of protein suggest that beans are protein-rich foods compared to beef, chicken, pork, and egg, which exhibit protein content of approximately 20, 21, 18, and 14 g/100 g, respectively [7]. Besides nutritive values, legumes are also the source of phenolics, especially isoflavones. A review by Singh et al. (2017) summarized phenolic contents and their relation with antioxidant activities, in which all beans were categorized into dry beans, lentils, chickpea, cowpea, pigeon pea, and peas [12]. Among all reported data, dry beans were mostly investigated with a wide range of total phenolic contents from 0.25 mg gallic acid equivalent (GAE)/g dry weight (DW) in kidney beans to 157.6 mg GAE/g DW in fava beans [13,14]. Similarly, antioxidant activities also varied depending on both internal (such as varieties) and external factors (such as extraction and antioxidant methods) [12].

Although the nutritive values and phytochemicals of several beans have been demonstrated, the rapid growth of bean cultivars from breeding renders a nutritional problem and health benefits compared to the original lineages. In Thailand, ten beans in three genera, including underutilized *Phaseolus lunatus* L. cultivars ‘38’, ‘47’, and ‘59’ and commonly consumed *Vigna umbellata* (Thunb.) Ohwi and H.Ohashi cultivar ‘107’, *Vigna angularis* (Wild.) Ohwi and Ohashi cultivar ‘108’, *Phaseolus vulgaris* L. cultivar ‘112’, *Glycine max* (L.) Merrill cultivars ‘SJ5’ and ‘CM60’, *Vigna mungo* (L.) Hepper cultivar ‘CN4’, and *Vigna radiata* (L.) Wilczek cultivar ‘CN84-1’, are usually employed as original lineages. However, data concerning the nutritive values and health-related properties of these beans in three genera are lacking, leading to selection difficulty in breeding new lineages within the same genera with specific nutritional and health benefits. Thus, the objective of this study was to determine the nutritive values (proximate analysis, vitamins, and minerals), phenolic contents, and antioxidant activities of ten beans cultivated in Thailand. To ensure the originality of the beans, these ten beans were identified and kept in the Genebank Research and Development Group, Biotechnology Research and Development Office, Ministry of Agriculture and Cooperatives, Thailand. The data gained from this research will be useful to encourage bean consumption as part of a healthy diet and support new breeding within the same genera with particular nutritional and health benefits.

## 2. Materials and Methods

### 2.1. Sample Selection, Preparation, and Extraction

**Figure 1:**
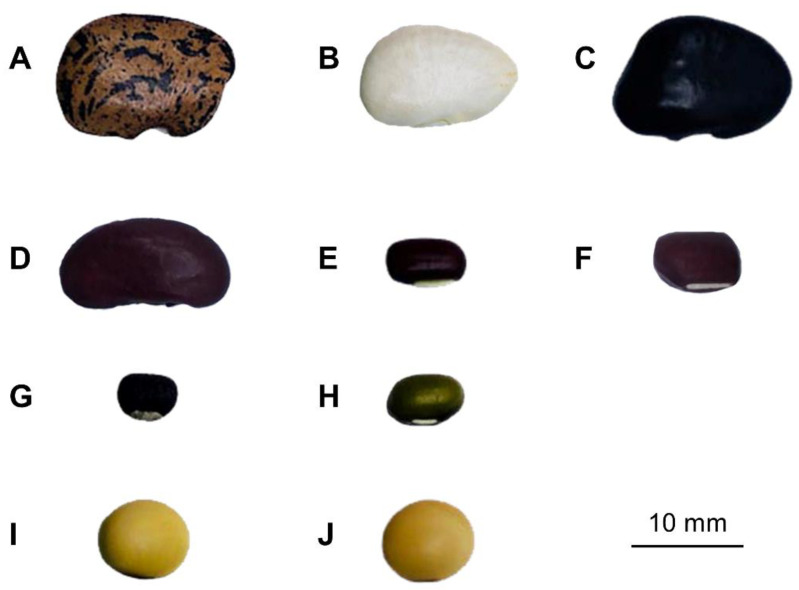
Illustration of bean seeds: *Phaseolus lunatus* L. cultivars (**A**) ‘38’, (**B**) ‘47’, and (**C**) ‘59’; (**D**) *Phaseolus vulgaris* L. cultivar ‘112’; (**E**) *Vigna umbellata* (Thunb.) Ohwi and H.Ohashi cultivar ‘107’; (**F**) *Vigna angularis* (Wild.) Ohwi and Ohashi cultivar ‘108’; (**G**) *Vigna mungo* (L.) Hepper cultivar ‘CN4’; (**H**) *Vigna radiata* (L.) Wilczek cultivar ‘CN84-1’; and *Glycine max* (L.) Merrill cultivars (**I**) ‘SJ5’ and (**J**) ‘CM60’.

Seeds of ten bean cultivars from Fabaceae (Leguminosae) family, Papilionoideae subfamily, were obtained from Genebank Research and Development Group, Biotechnology Research and Development Office, Department of Agriculture (DOA), Thailand. The appearance of 10 beans, namely *P. lunatus* L. cultivars ‘38’, ‘47’, and ‘59’; *P. vulgaris* L. cultivar ‘112’; *V. umbellata* (Thunb.) Ohwi and H.Ohashi cultivar ‘107’; *V. angularis* (Wild.) Ohwi and Ohashi cultivar ‘108’; *V. mungo* (L.) Hepper cultivar ‘CN4’; *V. radiata* (L.) Wilczek cultivar ‘CN84-1’; and *G. max* (L.) Merrill cultivars ‘SJ5’ and ‘CM60’, is shown in Figure 1, while morphological characteristics including seed size (width, length, and thickness) as measured by a 0.01mm/0.0005” digital Vernier (Protronics Co., Ltd., Pathum Thani, Thailand) and color as measured by a ColorFlex EZ spectrophotometer (Hunter Associates Laboratory, Reston, VA, USA) are shown in Supplementary Tables S1 and S2, respectively. Names and voucher specimens deposited at the Bangkok Herbarium (BK), Bangkok, Thailand, are shown in Table 1.

The samples were ground into fine powder using a Philips 600 W series grinder (Philips Electronic Co., Ltd., Jakarta, Indonesia) before separation into two groups. One was used for nutritional analysis (as shown in Section 2.2), and the other underwent ethanolic extraction for the determination of total phenolic contents (as shown in Section 2.3) and antioxidant activities (as shown in Section 2.4). For extraction, the powdery sample (1 g) was mixed with 70% (*v*/*v*) aqueous ethanol (20 mL) and shaken in a 30 °C WNE45 series water bath shaker (Memmert GmBh, Eagle, WI, USA) for 2 h. The supernatant was collected through centrifugation at 3,800× *g* for 15 min using a Hettich ROTINA 38R refrigerated centrifuge (Andreas Hettich GmbH, Tuttlingen, Germany) and filtered through a 0.45 µM polyethersulfone membrane syringe filter. The filtrates were kept at −20 °C until analysis.

### 2.2. Determination of Nutrients

Nutrients, including proximate compositions, vitamins, and minerals, were determined using standard protocols of the Association of Official Analytical Chemists (AOAC) [15]. All samples were analyzed in the Accredited Laboratory, complying with ISO/IEC 17025:2017, at the Institute of Nutrition, Mahidol University. The results per 100 g fresh weight (FW) are shown in Supplementary Tables S3–S5. To accurately compare the nutritive values among 10 bean cultivars in 3 genera, compositions per 100 g dry weight (DW) were calculated.

#### 2.2.1. Proximate Compositions

The proximate compositions, including moisture content, protein, fat, ash, carbohydrate, and energy, were determined as previously reported with modifications [16]. Briefly, moisture content was determined by drying samples in a Memmert UNE 500 hot-air oven (Eagle, WI, USA) at 100 ± 5 °C until a constant weight was obtained (AOAC Method No. 931.04). Total nitrogen was determined by the Kjeldahl method with digestion and distillation units (BÜCHI Corporation, New Castle, DE, USA) (AOAC Method No. 991.20), and nitrogen to protein content was calculated using a conversion factor of 6.25 for all samples, except for soybean, which used a conversion factor of 5.71 [17,18]. Total fat content was determined by acid hydrolysis and solvent extraction using Tecator Soxtec System HT 1043 (Foss Tecator, Hoganas, Sweden) (AOAC Method No. 922.06). Ash content was determined by incinerating organic matters in Carbolite CWF 1100 muffle furnace (Carbolite Gero Ltd., Hope, UK) at 550 ± 5 °C until the sample was free of carbon, cooled in a desiccator, and calculated for the amount of ash (AOAC Method No. 930.30). Total dietary fiber was determined by the enzymatic–gravimetric method (AOAC Method No. 985.29). Total carbohydrate was calculated using Equation (1), while energy was calculated by the Atwater factor using Equation (2):

Total carbohydrate = 100-moisture-protein-fat-ash(1)

Energy = (total carbohydrate × 4) + (protein × 4) + (total fat × 9)(2)

#### 2.2.2. Minerals

Ash residue was further used for the analysis of calcium, sodium, and potassium utilizing a Thermo S series flame atomic absorption spectrophotometer (AAS) (Thermo Electron Corporation, Cambridge, UK) (AOAC Method No. 985.35). Magnesium, iron, copper, and zinc contents were determined using an Optima 4200DV inductively coupled plasma optical emission spectroscope (ICP-OES) (PerkinElmer, Waltham, MA, USA) (AOAC Method No. 984.27).

#### 2.2.3. Vitamins

Determination of vitamin B1 (thiamin), vitamin B2 (riboflavin), and vitamin B3 (niacin) was performed using high-performance liquid chromatography (HPLC). Vitamins B1 and B2 were analyzed according to the in-house methods of INMU-TM-FCH-08 based on AOAC Method Nos. 942.23 and 970.65, respectively. The HPLC system is composed of an FP-920 fluorescence detector (JASCO International Co., Ltd., Tokyo, Japan), an LC-20AT pump (Shimadzu Scientific Instrument, Columbia, MD, USA), a Luna C18(2) 100A column (5 µm, 250 × 4.6 × 10^−3^ m, Phenomenex, Torrance, CA, USA), and an isocratic solvent system (50% (*v*/*v*) methanol) operating at a flow rate of 1.0 mL/min [19].

Vitamin B3 was determined by an in-house method based on AOAC (2019) 961.14. The HPLC system is composed of an 1100 series G1314B variable wavelength detector (VWD) UV detector (Agilent Technologies, Santa Clara, CA, USA), a 1200 series G1310A isocratic pump (Agilent Technologies, Santa Clara, CA, USA), a Luna C8(2) 100A column (5 µm, 250 × 4.6 × 10^−3^ m, Phenomenex, Torrance, CA, USA), and an isocratic solvent system (15% (*v/v*) methanol) operating at a flow rate of 1.0 mL/min [20,21].

### 2.3. Determination of Total Phenolic Contents

The Folin–Ciocalteu phenol assay was used to determine total phenolic contents (TPCs) as previously described by Sripum et al. (2017) [22], without any modification. Gallic acid (0–200 µg/mL) was employed as a standard, and the results were expressed as mg gallic acid equivalent (GAE)/g DW.

### 2.4. Determination of Antioxidant Activities

Three antioxidant assays, namely oxygen radical absorbance capacity (ORAC), ferric ion reducing antioxidant power (FRAP), and 2,2-diphenyl-1-picrylhydrazyl (DPPH) radical scavenging assays, were performed as previously described [23]. Briefly, sodium fluorescein and 2,2′-azobis(2-amidinopropane) dihydrochloride were the main reagents in the ORAC assay, while FRAP reagent containing acetate buffer, 2,4,6-tri(2-pyridyl)-*S*-triazine, and FeCl_3_·6H_2_O solution was used in the FRAP assay. The DPPH radical scavenging assay utilized a DPPH reagent. The extract concentrations in the antioxidant assays were in the range of a Trolox standard curve. Kinetic measurement at an excitation wavelength of 485 nm and an emission wavelength of 528 nm was for antioxidant determination of the ORAC assay, while end-point measurements at 600 and 520 nm were for FRAP and DPPH radical scavenging assays, respectively, using a Synergy HT 96-well UV–visible microplate reader with a Gen 5 data analysis software (BioTek Instruments, Inc., Winooski, VT, USA). The results were expressed as µmol Trolox equivalent (TE)/g DW.

### 2.5. Statistical Analysis

Experiments were performed in triplicate (*n* = 3), and results were expressed as mean ± standard deviation (SD). Statistical analysis was performed using a one-way analysis of variance (ANOVA), followed by Duncan’s multiple comparison test with significant differences at *p* < 0.05.

Principal component analysis (PCA) and hierarchical cluster analysis (HCA) of nutritive components, TPCs, and antioxidant activities were performed using XLSTAT (Addinsoft Inc., New York, NY, USA).

## 3. Results

### 3.1. Nutritive Values

#### 3.1.1. Proximate Compositions

The proximate compositions of 10 bean cultivars per 100 g DW are shown in Table 2, while the contents per 100 g FW are shown in Supplementary Table S3. The range of energy contribution was between 386.07 and 489.78 kcal/100 g DW. The major component in all samples was carbohydrate (33.17–77.39 g/100 g DW), with the distribution of dietary fiber from 12.56 to 23.23 g/100 g DW. Protein and fat contents varied between 16.62 and 40.40 and between 0.77 and 22.45 g/100 g DW, respectively, and ash content ranged from 3.51 to 5.62 g/100 g DW.

Among three genera of beans, *Glycine* beans contained the highest protein (accounting for 36–40% of total nutrients), followed by those in the *Vigna* (23–27%) and *Phaseolus* (17–23%) genera. Similar results were observed in fat contents, in which *Glycine* beans exhibited 21–22% fat, while only 1–2% fat was detected in *Vigna* and *Phaseolus* beans. In contrast, *Phaseolus* beans possessed higher carbohydrate contents (70–77%) than those in the *Vigna* (68–72%) and *Glycine* (33–35%) genera. Higher carbohydrate contents led to overall higher dietary fiber detected in *Phaseolus* beans than in the other two genera. Since protein and carbohydrate provide 4 kcal/g and fat provides 9 kcal/g (as indicated in Equation (2)), *Glycine* beans with higher protein and fat contents also provide higher energy than the other two genera.

Of the 10 cultivars considered, soybean (*G. max* (L.) Merrill) cultivar ‘SJ5’ exhibited the highest protein content, while another soybean cultivar in the same genus ‘CM60’ provided the highest fat content. Lima bean (*P. lunatus* L.) cultivar ‘47’ exhibited the highest carbohydrate content. However, black gram (*V. mungo* (L.) Hepper) cultivar ‘CN4’ provided the highest dietary fiber contents. Since fat provides the highest energy of 9 kcal/g, *G. max* (L.) Merrill cultivar ‘CM60’ possessed the highest energy.

#### 3.1.2. Vitamin Contents

Vitamins B1, B2, and B3 in 10 bean cultivars per 100 g DW are shown in Table 3, while the contents per 100 g FW are shown in Supplementary Table S4. The ranges of vitamins B1, B2, and B3 were 0.04–0.51, 0.02–0.11, and 0.51–1.55 mg/100 g DW, respectively. There was no clear trend of vitamin contents in different genera. For example, *G. max* (L.) Merrill cultivar ‘SJ5’ exhibited distinctly high vitamin B1 content, while another cultivar in the same genus ‘CM60’ exhibited a 6.4-fold lower content than other cultivars. However, among 10 cultivars, the highest vitamin B1 contents were observed in two beans from the *Phaseolus* genus, namely lima bean ‘38’ and red kidney bean ‘112’ (up to 12.8-fold higher than the others). Vitamin B2 was the highest in black gram ‘CN4’ (up to 5.5-fold higher), while red bean ‘107’ provided the highest vitamin B3 content (up to 3-fold higher).

#### 3.1.3. Mineral Contents

The mineral contents of 10 bean cultivars per 100 g DW are shown in Table 4, while the contents per 100 g FW are shown in Supplementary Table S5. All beans possessed predominantly potassium (accounting for 73–84% of all minerals), followed by magnesium (7–14%), calcium (2–14%), sodium (1–4%), iron (0.3–0.5%), and zinc (0.1–0.3%). No clear trend of potassium content in different cultivars was observed. Nevertheless, red kidney bean ‘112’ exhibited the highest potassium content (up to 1.7-fold higher than the others). All except two soybean cultivars, ‘SJ5’ and ‘CM60’, exhibited higher magnesium than calcium contents. Interestingly, these two cultivars exhibited the highest calcium contents among all cultivars (up to 7.6-fold higher than the others). Sodium content was also distributed differently among cultivars, with the highest contents being detected in azuki bean ‘108’ (up to 4.4-fold higher than the others). For microminerals, azuki bean ‘108’ exhibited the highest iron content (1.1–1.9-fold higher than the others), while two soybean cultivars exhibited the highest zinc content (1.5–2.3-fold higher).

### 3.2. Total Phenolic Contents and Antioxidant Activities

All beans exhibited TPCs in a range between 0.72 and 3.12 mg GAE/g DW (Table 5). Beans in the *Vigna* genus tended to exhibit higher TPCs than other genera, especially azuki bean ‘108’, which provided the highest TPCs (up to 4.3-fold higher than other cultivars). Corresponding to TPCs, beans in the *Vigna* genus also exhibited higher antioxidant activities than other genera. Azuki bean ‘108’ with the highest TPCs also exhibited the highest FRAP and DPPH radical scavenging activities compared to other cultivars (up to 6.6- and 7.1-fold higher). In addition, as one in the *Vigna* genus, mung bean ‘CN84-1’ also exhibited the highest ORAC activities, accounting for 1.3-11.9-fold higher activities than others.

### 3.3. Correlation Analysis by Principal Component Analysis (PCA) and Hierarchical Cluster Analysis (HCA)

Because the present study has voluminous informative data on nutritive values, TPCs, and antioxidant activities, it is difficult to analyze data with simple correlation analysis. To enhance the correlation analysis, we used PCA and HCA to analyze the data by using the mean of nutritive values (proximate compositions, vitamins, and minerals), TPCs, and antioxidant activities as determined by ORAC, FRAP, and DPPH radical scavenging assays from all ten bean cultivars.

**Figure 2:**
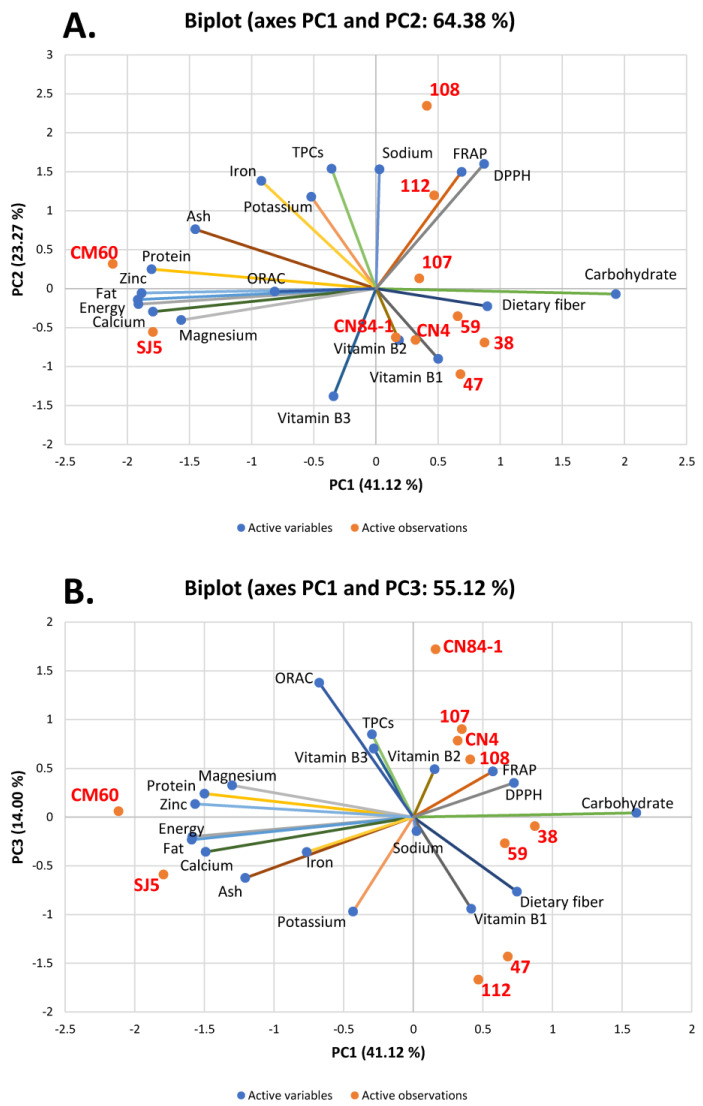
The biplot derived from principal component analysis (PCA). The mean values of (proximate compositions, vitamins, and minerals), total phenolic contents (TPCs), and antioxidant activities as being determined by oxygen radical absorbance capacity (ORAC), ferric ion reducing antioxidant power (FRAP), and 2,2-diphenyl-1-picrylhydrazyl (DPPH) radical scavenging assays of all ten bean cultivars were analyzed. (**A**) The plot between PC1 and PC2; (**B**) the plot between PC1 and PC3.

Figure 2A,B show the biplot analysis between variables (proximate compositions, vitamins, and minerals), TPCs, and antioxidant activities determined by ORAC, FRAP, and DPPH radical scavenging assays and observations (bean cultivars). Three axes denoted as PC1, PC2, and PC3 covered 78.38% (PC1 = 41.12%, PC2 = 23.27%, and PC3 = 14.00%) of all analyzed data, indicating a good representation of the correlation analysis. PC1 explained 41.12% of variance containing energy, protein, fat, carbohydrate, ash, calcium, magnesium, and zinc. PC2 explained 23.27% of variance containing vitamin B2, vitamin B3, sodium, potassium, iron, TPCs, and antioxidant activities determined by the FRAP and DPPH radical scavenging assays. While fiber, vitamin B1, and ORAC activities were presented in PC3, the PCA analysis illustrated that two soybeans, ‘SJ5’ and ‘CM60’, were high in nutritive values, including energy, protein, fat, ash, calcium, magnesium, and zinc (Figure 2A,B) because these two cultivars were located close to the mentioned variables. Red kidney bean ‘112’ and azuki bean ‘108’ were positioned close to sodium as well as FRAP and DPPH radical scavenging activities, suggesting high values in these beans. Interestingly, Figure 2B suggested that red rice bean ‘107’ was in close proximity with TPCs and vitamin B3, while mung bean ‘CN84-1’ was close to ORAC activities, suggesting that these cultivars exhibited high contents of such parameters. In summary, the PCA data indicated that among ten bean cultivars, soybean ‘CM60’ was the most promising bean regarding high nutritive values, minerals, vitamins, TPCs, and antioxidant activities.

**Figure 3:**
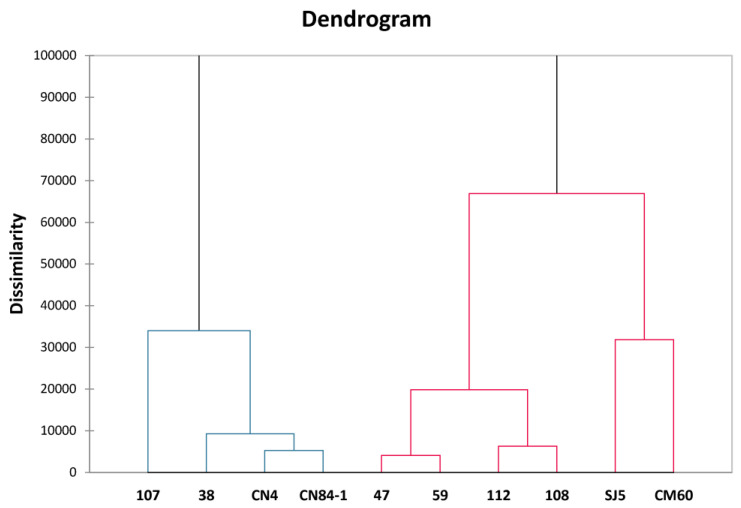
The dendrogram obtained from the mean values (proximate compositions, vitamins, and minerals), total phenolic contents (TPCs), and antioxidant activities as being determined by oxygen radical absorbance capacity (ORAC), ferric ion reducing antioxidant power (FRAP), and 2,2-diphenyl-1-picrylhydrazyl (DPPH) radical scavenging assays of all beans with hierarchical cluster analysis (HCA).

To study the clustering of all tested beans, we further analyzed the mean values (proximate compositions, vitamins, and minerals), TPCs, and antioxidant activities determined by ORAC, FRAP, and DPPH radical scavenging assays of all beans with hierarchical cluster analysis (HCA) (Figure 3). The results also confirmed the data evaluated by PCA. Two soybeans, ‘SJ5’ and ‘CM60’, were clustered together, suggesting a close relationship between them. Here, we also observed some relationships missing from PCA, such as red kidney bean ‘112’ and azuki bean ‘108’, as well as lima beans, ‘47’ and ‘59’.

## 4. Discussion

Ten bean cultivars can be divided into (1) the genus *Phaseolus* including *P. lunatus* L. or lima bean cultivars ‘38’, ‘47’, and ‘59’, and *P. vulgaris* L. or red kidney bean cultivar ‘112’; (2) the genus *Vigna* including *V. umbellata* (Thunb.) Ohwi and H.Ohashi or red bean cultivar ‘107’, *V. angularis* (Wild.) Ohwi and Ohashi or azuki bean cultivar ‘108’, *V. mungo* (L.) Hepper or black gram cultivar ‘CN4’, and *V. radiata* (L.) Wilczek or mung bean cultivar ‘CN84-1’; and (3) the genus *Glycine* including *G. max* (L.) Merrill or soybean cultivars ‘SJ5’ and ‘CM60’. This is the first report to detail the nutritive values, total phenolic contents, and antioxidant activities of ten bean cultivars collected in Genebank, Department of Agriculture (DOA), Thailand. The results demonstrated that beans in the *Glycine* genus tended to exhibit high protein and fat contents, leading to higher energy than other genera. These *Glycine* beans also exhibited high calcium content. On the contrary, *Phaseolus* beans tended to exhibit high carbohydrate contents, resulting in high dietary fiber. In addition, two bean cultivars in the *Phaseolus* genus, namely lima bean ‘38’ and red kidney bean ‘112’, contained the highest amount of vitamin B1, while the latter also exhibited the highest potassium content. Different bean cultivars in the *Vigna* genus possess distinct nutritional compositions. Black gram ‘CN4’ and red rice bean ‘107′ exhibited the highest vitamin B2 and B3 contents, respectively, while sodium and iron were the highest in azuki bean ‘108’. Interestingly, *Vigna* beans tended to exhibit higher TPCs than other genera, leading to high antioxidant activities.

### 4.1. Nutritional Compositions

Soybeans are distinctly high in energy, protein, and fat. Based on Thai Recommended Daily Intakes (Thai RDIs) [24,25], 100 g of our soybeans provides protein and fat at up to 76% and 32%, respectively, of Thai RDIs. Although the fiber contents of soybeans were the lowest among the ten cultivars (1.4 folds lower) in this study, 100 g of soybeans provide a significant amount of fiber (64% Thai RDI). The Dietary Guidelines for Americans (DGAs 2020–2025) recommend a bean, pea, and lentil intake of 1.5 cups per week or equivalent to 37.5 g of cooked legumes per day for a healthy U.S.-style dietary pattern [26]. Since 1 cup of raw beans and 1 cup of cooked beans are equivalent to 60 and 175 g, respectively [27], 37.5 g of cooked beans is equivalent to 12.85 g of raw beans. Based on our information, consuming this amount of soybeans per day provides 4.7 g of protein, 2.0 g of fiber, 29.20 mg of calcium, 165.20 mg of potassium, 0.82 mg of iron, 0.032 mg of vitamin B1, and 0.165 mg of vitamin B3; these values are higher than those provided by consuming half a boiled egg (25 g) [28]. Protein is a macronutrient necessary for maintaining normal body growth and development [29]. The high protein content in soybean is a unique characteristic for food applications and products sought by vegetarians, vegans, and those searching for alternatives to meat [30]. Soybean is known as oilseed and is used extensively in the production of cooking oil with a large portion of polyunsaturated fats (57.39%), mainly omega-6 (44.44%) [28], which is a healthier choice compared to high saturated fat [31]. Supplementary Table S6 shows that soybeans in the U.S. Department of Agriculture (USDA), Food Data Central (FDC, FDC ID 174270), and Thai Food Composition databases (FCD, Food code C33) had similar levels of energy, protein, fat, carbohydrate, and ash to those found in our study [28,32]. However, fiber content from Thai FCD was 1.4 times higher than ours, and that from the USDA was 1.7 times lower than ours. In addition, vitamins B1, B2, and B3 and iron in both databases were up to 18, 32, 2, and 2.6 times higher, respectively, while soybean ‘CM60’ had extremely higher sodium (22 times) when compared with the USDA database [28,32].

Beans in the *Phaseolus* genus contained the highest amount of carbohydrates. In agreement with Alcázar-Valle et al. (2020), carbohydrate was the major component in lima beans, while fat was found at a low level [33]. The same characteristics were observed in red rice bean, azuki bean, red kidney bean, black gram, and mung bean, in descending order. Our data suggested that lima beans are good sources of fiber, providing 69–80% of Thai RDI. Even though protein contents of lima beans were in the bottom three among ten cultivars, they provide 30–41% Thai RDI for protein. In previous research, lima bean flour (red, white, and brown) exhibited similar fat content (0.96–0.97 g/100 g FW), 1.5–2 times higher protein (27.80–29.55 g/100 g FW), and extremely lower levels of fiber (6–12 times lower) (crude fiber 1.65–3.40 g/100 g FW) [34]. The variations could be due to different bean cultivars, genotypes, planting locations, environments, sample preparations, and analytical methods. Since we determined the total dietary fiber using the enzymatic–gravimetric method, the previous study used the crude fiber method with acid–base digestion, which usually yielded lower fiber values since acid and base can dissolve parts of fiber, and the remains are mainly lignin and cellulose [35,36]. Supplementary Table S7 demonstrated nutritive values of lima beans in USDA (FDC ID 174252) [32] and the FAO/INFOODS Global database for pulses on a dry matter basis (PulsesDM) (Food ID PHL001_DM), which compiled data from published papers and food composition databases from Australia, Denmark, Thailand, the United Kingdom, and the USA [37]. Both databases showed a similar proportion of proximate compositions to our study. Vitamin B1 levels were similar to those found in lima bean ‘38’, while vitamin B2 contents in the two databases were 7 times higher than ours [32,37].

Red kidney bean (*P. vulgaris*) provided a high amount of vitamin B1, equivalent to 31% Thai RDI. Supplementary Table S8 showed that proximate contents, vitamin B1, and minerals of red kidney beans in PulseDM (Food ID PHV004_DM), USDA (Food ID 173744), and Thai FCD (Food code C18) [28,32,37] were similar to those found in our study with the exception of vitamins B2 and B3, for which databases showed 7–50 and 5–6 times higher contents, respectively. In addition, sodium contents in the databases were 4–5 times lower than ours [28,32,37].

For red beans or red rice beans (*V. umbellata*, previously *P. calcaratus*), most proximate compositions of ‘107’ cultivar were comparable to those of a previous study in India (rice bean, JRC-08-10) [38]. However, the amount of crude fiber (6.06 g/100 g FW) was much lower when compared with our study [38]. This could be due to the factors mentioned above. In Supplementary Table S9, the proximate compositions and minerals from Thai FCD (Food code C16) and PulseDM (Food ID VIU002_DM) were comparable to those of our red rice bean ‘107’ [28,37]. Regardless of the highest vitamin B3 and magnesium contents in the ‘107’ cultivar, the values of rice beans from PulseDM were slightly higher (2.4 and 1.3 times higher), while vitamins B1 and B2, calcium, and sodium from the databases were 8, 10, 10, and 2.5 times higher, respectively [28,37]. Despite azuki bean ‘108’ having fiber and protein contents in the seventh and third ranks, respectively, it provides fiber at 66% and protein at 50% of Thai RDIs per 100 g FW. A previous study reported that the azuki bean exhibited 3.5–3.7% moisture, 20.3–21.5% protein, and 1.3–1.5% fat [39]. Our study reported twice the moisture content and slightly higher protein contents (1.2 times higher), while our fat content was 2 times lower. The differences might be due to cultivars, locations, weather and climate, storage conditions, moisture content, and analysis methods. The proximate compositions and most minerals of azuki bean ‘108’ agreed with those reported in PulseDM (Food ID VIA001_DM) and USDA (FCD ID 173727) databases [32,37]. However, both databases (unknown seed coat color) demonstrated extremely high contents of vitamins B1 and B2 (13 times higher). In contrast, databases showed 20 and 13 times lower magnesium and sodium, respectively, than our azuki bean ‘108’ sample [32,37]. Although they have the same scientific name, the discrepancy in nutritive values might be due to the different coat colors, cultivars, and external environmental factors.

Vitamin B2 was found to be the highest in black gram, while mung bean contained 2.8 times lower vitamin B2 content. Dietary fiber contents of the two beans were surprisingly different. Black gram provided the highest fiber content (23.23 g/100 g DW). In contrast, mung bean contained the lowest level of fiber (1.8 times lower). Thus, black gram is a great source of fiber, and 100 g FW provides up to 86% of Thai RDI. As shown in Supplementary Table S10, all nutritive values of black gram ‘CN4’ were comparable to those of mung beans from PulseDM (Food ID VIM001_DM) and USDA (FDC ID 174259) databases [32,37]. When compared with mung bean ‘CN84-1’, Thai FCD (Food code C15), USDA (FCD ID 174256), and PulseDM (Food ID VIR001_DM) databases contained 6.5–7 times higher vitamin B2 content, and Thai FCD (Food code C15) demonstrated higher fiber, vitamin B1, and sodium (2.3, 5.5, and 3.5 times higher, respectively) [28,32,37].

### 4.2. Total Phenolic Contents and Antioxidant Potentials

Common beans (*P. vulgaris* L.) are in a highly diverse group consisting of a wide variety of cultivars such as kidney beans, navy beans, black beans, red beans, cranberry beans, and pinto beans. It was summarized in a review by Ganesan and Xu (2017) that most phenolics are located in seed coats, especially anthocyanins in colored beans [40]. Beans in this species possess phenolic content at approximately 11% of total weight or 145 mg/g [41]. It was previously reported that ten common beans (pinto, great Northern, navy, black, dark red kidney, light red kidney, red Mexican, cranberry, pink, and alubia) extracted with acidic methanol exhibited total phenolic acid contents in the range of 0.19–0.48 mg/g [42]. In addition, six common beans (black, cranberry, dark red kidney, light red kidney, navy, and pinto) extracted with 80% (*v*/*v*) aqueous ethanol exhibited TPCs of 3.28–16.61 mg catechin equivalent/g [43]. Methanolic extracted red beans exhibited TPCs of 6.87 mg GAE/g [44], while black beans extracted with an acetone/water/acetic acid mixture exhibited a TPC of 9.70 mg GAE/g [45]. Red kidney beans extracted with acidic ethanol also exhibited a TPC of 27.1 mg GAE/g [46]. Compared to our results on red kidney bean ‘112’ with a TPC of 2.0 mg GAE/g DW, different results compared to the literature might be due to the different extraction methods and standards used in the assay. Another *Phaseolus* genus, lima bean (*P. lunatus* L.), also possesses diverse seed coat colors such as white, brown, light brown, red, pink, and black [47]. It was previously found that eighteen cultivars of lima bean extracted with the mixture of acetone–water–acetic acid (70:29.5:0.5 *v*/*v*) exhibited TPCs in the range of 0.1–7.6 mg GAE/g, while ones with a lighter seed coat (white) exhibited lower TPCs than the ones with darker seed coat colors (such as black and red) [47]. These data corresponded to our results, in which lima bean ‘47’ with white seed coat provided lower TPCs than lima beans ‘38’ and ‘59’, with brown and black seed coats, respectively. Likewise, rice bean (*V. umbellata*) also possesses different seed coat colors such as black, green, yellow, and red [48]. Aqueous ethanolic extracts of 13 varieties of rice bean (unknown seed coat color) exhibited TPCs of 3.27–6.43 mg GAE/g [49]. Similar results were observed in our experiment, in which red rice bean ‘107’ exhibited a TPC of 2.4 mg GAE/g DW. Interestingly, rice bean and azuki bean (*V. angularis*) possess high similarity in terms of nucleotide sequence, leading them to resemble proteins and isozymes [50,51], and, thus, similar phenolic contents. Fifty-eight varieties of azuki bean exhibited TPCs of 1.45–3.97 mg GAE/g [52], which are in the range of TPC of our azuki bean ‘108’ (TPC of 3.1 mg GAE/g DW). Our TPC result (2.09 mg GAE/g DW) for black gram ‘CN4’ (*V. mungo*) also corresponded to a previous report which stated that black gram extracted with 80% (*v*/*v*) aqueous acetone and 80% (*v*/*v*) aqueous ethanol exhibited TPCs of 3.82 and 2.11 mg GAE/g DW, respectively [53]. In addition, 56 mung bean varieties (*V. radiata*) analyzed by HPLC exhibited phenolic contents ranging from 1.61 to 3.46 mg/g DW [54]. Even though a different method was used in our study, the results were similar, in which a TPC of 1.92 mg GAE/g DW was detected in our mung bean ‘CN84-1’. The results of our soybeans (*G. max*), ‘SJ5’ and ‘CM60’, with TPCs of 1.60 and 2.54 mg GAE/g DW, respectively, were similar to previous literature that reported soybean extracted with 80% (*v*/*v*) aqueous methanol exhibited a TPC of 1.08 mg GAE/g DW [55], while 20 soybean cultivars extracted with 70% (*v*/*v*) aqueous acetone exhibited TPCs of 2.70-4.88 mg catechin equivalent/g DW [56].

It was previously suggested that TPCs possessed a strong correlation with antioxidant activities [57]. In the *Phaseolus* genus, 18 lima bean cultivars exhibited antioxidant activities in a range of 2.6–94.4%, as determined by the DPPH radical scavenging assay [47]. In addition, red kidney beans extracted with 80% (*v*/*v*) aqueous ethanol exhibited DPPH radical scavenging activity of 12.43% [43], while its acidic-ethanolic extract also exhibited DPPH radical scavenging activity of 66.15% [46]. As for the Vigna genus, methanolic extracted red rice bean exhibited DPPH radical scavenging activity with a half-maximal effective concentration (EC_50_) of 0.49 mg/mL [44], while aqueous ethanolic extracts of 13 varieties of rice bean (unknown seed coat color) exhibited DPPH radical scavenging activity of 39.87–46.40 µM TE/g [49]. In addition, 58 varieties of azuki bean exhibited DPPH radical scavenging activity of 78.39–88.39 µM TE/g [52], while methanolic extracts of two azuki bean cultivars exhibited DPPH radical scavenging activity with EC_50_ values of 148.83–243.41 mg/mL [58]. Black gram extracted with 80% (*v*/*v*) aqueous acetone exhibited DPPH radical scavenging activity with a half-maximal inhibitory concentration (IC_50_) of 5.36 mg of GAE [53], and mung bean extracted with acidic ethanol also exhibited DPPH radical scavenging activity of 59.01% [46]. In the *Glycine* genus, it was previously found that 20 soybean cultivars extracted with 70% (*v*/*v*) aqueous acetone exhibited DPPH radical scavenging activity of 22.87–48.17% [56]. The information indicated that these beans exhibited antioxidant activities. However, it was difficult to compare those results with ours due to different detection methods, standards used, and expression units.

There are several other non-nutrient bioactive compounds in plants; examples include oligosaccharides, phytic acids, trypsin inhibitors, tannins, saponins, lectins, and oxalate in legumes [59]. They are chemicals that play a role in the natural defense mechanism of plants against pathogens, insects, and pests, and the amounts vary among different plant genotypes and growing environments [60,61]. For example, raw black grams from India contained a low amount of tannin (0.5 mg/g DW) and phytic acid (5.1 mg/g DW) but demonstrated high trypsin inhibitor activity (120 TIU/g DW) [62]. Interestingly, many non-nutrient bioactive compounds also demonstrated health-promoting properties. Oligosaccharides may induce flatulence but are also considered prebiotics. Both phytic acids and phenolic compounds can reduce protein and carbohydrate digestibility and mineral bioavailability; however, they demonstrate antioxidant activities and anti-cancer properties [59,63].

Traditional food preparation methods, including dehulling, milling, soaking, germination, fermentation, and cooking, can reduce the non-nutrient bioactive compounds [60,64]. Shimelis and Rakshit (2007) demonstrated that raw kidney beans from Africa contained tannin (5.37 mg/g DW), phytic acid (23.51 mg/g DW), and trypsin inhibitor activity (20.89 TIU/g DW), and water soaking for 12 h followed by boiling for 35 min could reduce 70% of tannin and 65% of phytic acid, respectively, while sprouting for 48 h followed by autoclaving for 30 min completely removed non-nutrient bioactive compounds [65]. Hence, a combination of methods could help reduce most of the non-nutrient bioactive compounds [65]. Food preparation and processing methods can affect the availability of nutrients and bioactive compounds [66,67]. As shown by Huertas et al. (2022), 40% of iron and zinc were lost in the cooking water when raw beans (*P. vulgaris*) were prepared by boiling, while steaming is more potent in enhancing mineral availability [67]. According to the INMUCAL-Nutrients version 4.0 software, when comparing 100 g FW nutritive values of boiled and steamed beans to raw beans, the conversion factors for cooking methods were 0.39 and 0.44, respectively. Therefore, at 100 g FW, boiled and steamed beans contained 2.56 and 2.27 times lower nutritive values than those of raw beans; the same cooking factors are applied to mung bean (code 03024), red bean (code 03029), and soybean (code 03027). While for red kidney bean (code 03072), the conversion factors for boiling and steaming methods were 0.42 and 0.51, thus having 2.38 and 1.96 times lower nutrients than a raw red kidney bean, respectively. Not only the traditional preparation and cooking methods but also the emerging food processing techniques (i.e., microwave heating, infrared heating, extrusion cooking, etc.) can be tailored for the purpose of each pulse type to gain the desired nutritional and non-nutritional profiles in the final products [66].

The strength of this study is the standardized methods and protocols used, including the examination of nutritional compositions based on the AOAC; the Folin–Ciocalteu phenol assay to determine TPCs; and three antioxidant assays, namely ORAC, FRAP, and DPPH radical scavenging assays. Moreover, we analyzed the nutritive components, TPCs, and antioxidant activities using the PCA and HCA, which are statistical methods widely used to simplify the complexity of high-dimensional data to identify data patterns and express data in a way that can highlight their similarities and differences. In addition, the study approach was based on comparing bean cultivars belonging to three different genera: *Phaseolus*, *Vigna*, and *Glycine*. Legumes can be divided into two main categories: (1) oilseed legumes which provide a high amount of fat, including peanuts and soybeans, and (2) non-oilseed legumes (undried legumes and dried legumes). The dried legumes are termed “pulses”, which include beans, peas, and lentils [68,69]. Pulses are known for their nutrient density and health benefits, and beans are the major pulses consumed worldwide [68,69]. Therefore, we focused on ten original lineage beans in Thailand, eight of which were dried beans from the *Phaseolus* and *Vigna* genera as the representatives of non-oilseed legumes, including three types of the underutilized lima beans, three types of commonly consumed red beans, and commonly consumed mung bean and black gram. Moreover, we chose two types of commonly consumed soybeans from the *Glycine* genus as representatives of oilseed legumes. These ten original lineage beans were the major cultivars being promoted for cultivation in Thailand by the Department of Agriculture. Hence, the results of this research could be used for encouraging bean consumption as part of a healthy diet and used in other health promotion-related aspects in the future. Although this study was carefully designed and performed, limitations are that the antinutrient analysis and the effect of sample treatment other than grinding were not within the scope of this study. Since the antinutrient types and amounts can be varied according to plant genotypes and food preparation methods, future studies should incorporate the measurement of major antinutrients and the effect of food processing.

## 5. Conclusions

This study is the first to demonstrate the nutritive values, total phenolic contents, and antioxidant activities of ten bean cultivars from Genebank, Department of Agriculture (DOA), Thailand. Comparing among cultivars, soybean cultivars ‘SJ5’ and ‘CM60’ were found to be good sources of energy, protein, fat, calcium, magnesium, and zinc, while lima bean cultivars ‘38’, ‘47’, and ‘59’ and red kidney bean cultivar ‘112’ provided high levels of carbohydrates and dietary fiber. In addition, lima bean cultivar ‘38’ also exhibited high vitamin B1 content, while red kidney bean cultivar ‘112’ also provided high levels of potassium and vitamin B1. Black gram cultivar ‘CN4’ was high in dietary fiber and vitamin B2, and red rice bean cultivar ‘107’ exhibited high vitamin B3 content. Azuki bean ‘108’ exhibited the highest sodium, iron, TPCs, and antioxidant activities as determined by FRAP and DPPH radical scavenging assays, while high ORAC activities were detected in mung bean ‘CN84-1’. Therefore, the commonly consumed and underutilized Thai beans are nutrient-dense foods providing a good source of protein, fiber, vitamins, minerals, phenolic compounds, and antioxidants. The data would support the consumption of beans and their products as part of a healthy diet and could be used for incorporating the recommendation of legume consumption in the dietary guideline of Thailand. In addition, they will also be useful for new bean breeding within the same genera with specific nutrient requirements or high phenolic contents and antioxidant activities. Based on these data, for example, if one wants to develop a new bean breed with high protein content, beans in the *Glycine* genus would be a good choice of selection. The results might yield a new breed with higher protein content than its parents. Future studies are necessary to identify the bioactive compounds and their biological properties, antinutrients, effect of processing, and health benefits in promoting production and sustainable consumption for health promotion and disease prevention.

## Figures and Tables

**Table 1:** The scientific names, English common names, local Thai names, cultivars, and voucher specimens (Bangkok Herbarium (BK) numbers) of bean samples.

Scientific Name	English Common Name	Local Thai Name	Cultivar	Voucher Specimen (BK No.)
*Phaseolus lunatus* L.	Lima bean	Thua Lima	38	083065
		Thua Kao	47	083066
		Thua Boy	59	083064
*Phaseolus vulgaris* L.	Red kidney bean	Thua Daeng Luang	112	083063
*Vigna umbellata* (Thunb.) Ohwi and H.Ohashi	Red bean, rice bean	Thua Nio Nang Daeng	107	083067
*Vigna angularis* (Wild.) Ohwi and Ohashi	Azuki bean	Thua Azuki	108	083062
*Vigna mungo* (L.) Hepper	Black gram, mungo bean	Thua Khiao Phio Dam	CN4	083061
*Vigna radiata* (L.) Wilczek	Mung bean	Thua Khiao Phio Man	CN84-1	083072
*Glycine max* (L.) Merrill	Soybean	Thua Lueang	SJ5	083060
			CM60	083057

**Table 2:** Proximate compositions of ten bean cultivars (per 100 g dry weight).

Cultivar	Energy (kcal)	Protein (g)	Fat (g)	Carbohydrate (g)	Dietary Fiber (g)	Ash (g)
38	392.28 ± 0.54 ^c^	22.47 ± 0.07 ^g^	1.27 ± 0.12 ^d^	72.75 ± 0.19 ^c^	21.34 ± 0.39 ^c^	3.51 ± 0.01 ^i^
47	391.47 ± 1.39 ^cd^	16.62 ± 0.25 ^i^	1.72 ± 0.28 ^c^	77.39 ± 0.54 ^a^	21.93 ± 0.17 ^b^	4.28 ± 0.00 ^f^
59	389.75 ± 0.39 ^d^	19.20 ± 0.27 ^h^	1.33 ± 0.55 ^d^	75.25 ± 0.25 ^b^	19.21 ± 0.28 ^f^	4.22 ± 0.35 ^f^
112	386.39 ± 1.60 ^e^	23.00 ± 0.03 ^f^	1.38 ± 0.26 ^cd^	70.48 ± 0.14 ^e^	20.93 ± 0.23 ^d^	5.13 ± 0.09 ^c^
107	386.76 ± 0.12 ^e^	22.59 ± 0.07 ^g^	1.04 ± 0.02 ^de^	71.75 ± 0.08 ^d^	19.95 ± 0.01 ^e^	4.62 ± 0.01 ^d^
108	386.07 ± 0.60 ^e^	26.95 ± 0.11 ^c^	0.77 ± 0.12 ^e^	67.84 ± 0.01 ^g^	17.75 ± 0.31 ^g^	4.44 ± 0.01 ^e^
CN4	390.96 ± 0.34 ^cd^	25.42 ± 0.22 ^e^	1.10 ± 0.12 ^de^	69.83 ± 0.40 ^f^	23.23 ± 0.02 ^a^	3.64 ± 0.06 ^h^
CN84-1	391.37 ± 0.32 ^cd^	26.30 ± 0.05 ^d^	1.40 ± 0.05 ^cd^	68.39 ± 0.09 ^g^	12.56 ± 0.14 ^j^	3.91 ± 0.01 ^g^
SJ5	485.32 ± 0.32 ^b^	40.40 ± 0.05 ^a^	21.22 ± 0.11 ^b^	33.17 ± 0.20 ^i^	16.59 ± 0.05 ^i^	5.20 ± 0.05 ^b^
CM60	489.78 ± 2.41 ^a^	36.38 ± 0.24 ^b^	22.45 ± 0.48 ^a^	35.24 ± 0.71 ^h^	17.06 ± 0.18 ^h^	5.62 ± 0.01 ^a^

All data are expressed as mean ± standard deviation (SD) of triplicate experiments (*n* = 3). Different superscript letters indicate significantly different contents of the same proximate composition in different bean cultivars (*p* < 0.05) using one-way analysis of variance (ANOVA) and Duncan’s multiple comparison test.

**Table 3:** Vitamin contents of ten bean cultivars (per 100 g dry weight).

Cultivar	Vitamin B1 (mg)	Vitamin B2 (mg)	Vitamin B3 (mg)
38	0.51 ± 0.02 ^a^	0.03 ± 0.00 ^c^	1.31 ± 0.01 ^c^
47	0.37 ± 0.02 ^c^	0.03 ± 0.01 ^c^	1.42 ± 0.01 ^b^
59	0.36 ± 0.01 ^c^	0.03 ± 0.00 ^c^	1.34 ± 0.01 ^bc^
112	0.50 ± 0.01 ^a^	0.03 ± 0.01 ^c^	0.51 ± 0.07 ^e^
107	0.14 ± 0.02 ^e^	0.03 ± 0.00 ^c^	1.55 ± 0.06 ^a^
108	0.04 ± 0.00 ^g^	0.02 ± 0.00 ^d^	0.96 ± 0.15 ^d^
CN4	0.44 ± 0.02 ^b^	0.11 ± 0.00 ^a^	1.39 ± 0.04 ^bc^
CN84-1	0.16 ± 0.02 ^d^	0.04 ± 0.00 ^b^	1.35 ± 0.01 ^bc^
SJ5	0.45 ± 0.02 ^b^	0.03 ± 0.00 ^c^	1.43 ± 0.02 ^b^
CM60	0.07 ± 0.01 ^f^	0.03 ± 0.00 ^c^	1.30 ± 0.01 ^c^

All data are expressed as mean ± standard deviation (SD) of triplicate experiments (*n* = 3). Different superscript letters indicate significantly different contents of the same vitamin in different bean cultivars (*p* < 0.05) using one-way analysis of variance (ANOVA) and Duncan’s multiple comparison test.

**Table 4:** Mineral contents of ten bean cultivars (per 100 g dry weight).

Cultivar	Macromineral (mg)				Micromineral (mg)	
	Calcium	Sodium	Potassium	Magnesium	Iron	Zinc
38	86.40 ± 1.15 ^f^	33.34 ± 8.55 ^c^	990.13 ± 39.68 ^f^	115.46 ± 0.13 ^g^	4.30 ± 0.45 ^ef^	1.93 ± 0.04 ^f^
47	94.87 ± 0.14 ^e^	28.36 ± 0.59 ^cd^	1336.65 ± 39.39 ^c^	138.76 ± 1.58 ^d^	4.75 ± 0.63 ^e^	2.05 ± 0.07 ^e^
59	100.47 ± 0.51 ^cd^	19.59 ± 2.14 ^de^	1375.48 ± 27.10 ^c^	128.91 ± 1.58 ^e^	4.12 ± 0.16 ^f^	2.03 ± 0.00 ^e^
112	104.12 ± 1.78 ^c^	53.48 ± 9.95 ^b^	1517.36 ± 17.67 ^a^	118.95 ± 0.23 ^f^	7.00 ± 0.09 ^b^	2.38 ± 0.01 ^d^
107	31.97 ± 0.24 ^h^	16.75 ± 1.40 ^e^	1150.33 ± 12.64 ^e^	174.83 ± 0.30 ^b^	5.39 ± 0.28 ^d^	2.90 ± 0.01 ^b^
108	71.53 ± 1.29 ^g^	73.97 ± 8.54 ^a^	1449.51 ± 25.10 ^b^	114.79 ± 0.25 ^g^	7.56 ± 0.33 ^a^	2.33 ± 0.02 ^d^
CN4	117.15 ± 1.68 ^b^	45.98 ± 4.30 ^b^	915.34 ± 31.61 ^g^	170.20 ± 0.58 ^c^	6.04 ± 0.25 ^c^	2.95 ± 0.00 ^b^
CN84-1	96.85 ± 2.48 ^de^	21.54 ± 0.65 ^de^	972.51 ± 6.37 ^fg^	139.78 ± 0.57 ^d^	3.97 ± 0.13 ^f^	2.55 ± 0.02 ^c^
SJ5	241.39 ± 0.74 ^a^	20.63 ± 1.69 ^de^	1249.61 ± 5.82 ^d^	173.74 ± 1.27 ^b^	6.91 ± 0.26 ^b^	4.41 ± 0.07 ^a^
CM60	242.17 ± 8.14 ^a^	47.61 ± 8.74 ^b^	1487.96 ± 84.79 ^ab^	224.81 ± 3.67 ^a^	6.63 ± 0.27 ^b^	4.31 ± 0.15 ^a^

All data are expressed as mean ± standard deviation (SD) of triplicate experiments (*n* = 3). Different superscript letters indicate significantly different contents of the same mineral in different bean cultivars (*p* < 0.05) using one-way analysis of variance (ANOVA) and Duncan’s multiple comparison test.

**Table 5:** Total phenolic contents (TPCs) and antioxidant activities of ten bean cultivars.

Cultivar	TPCs (mg GAE/g DW)	Antioxidant Activities		
		ORAC Assay (µmol TE/g DW)	FRAP Assay (µmol TE/g DW)	DPPH Radical Scavenging Assay (µmol TE/100 g DW)
38	1.60 ± 0.07 ^g^	114.95 ± 6.65 ^f^	8.03 ± 0.34 ^d^	0.48 ± 0.03 ^d^
47	0.72 ± 0.04 ^h^	21.00 ± 1.90 ^i^	2.74 ± 0.11 ^h^	0.15 ± 0.01 ^g^
59	1.87 ± 0.08 ^f^	100.89 ± 5.25 ^g^	7.38 ± 0.32 ^e^	0.55 ± 0.05 ^c^
112	1.99 ± 0.16 ^e^	59.42 ± 5.40 ^h^	8.53 ± 0.59 ^c^	0.78 ± 0.07 ^b^
107	2.43 ± 0.10 ^c^	128.46 ± 11.36 ^e^	14.38 ± 0.76 ^b^	0.59 ± 0.05 ^c^
108	3.12 ± 0.10 ^a^	139.15 ± 12.21 ^d^	18.00 ± 0.60 ^a^	1.06 ± 0.08 ^a^
CN4	2.09 ± 0.06 ^d^	177.54 ± 10.72 ^c^	5.12 ± 0.17 ^f^	0.54 ± 0.04 ^c^
CN84-1	1.92 ± 0.10 ^ef^	249.95 ± 5.53 ^a^	5.19 ± 0.08 ^f^	0.48 ± 0.04 ^d^
SJ5	1.60 ± 0.05 ^g^	138.27 ± 3.48 ^d^	4.31 ± 0.19 ^g^	0.28 ± 0.02 ^e^
CM60	2.54 ± 0.08 ^b^	197.83 ± 5.64 ^b^	4.04 ± 0.22 ^g^	0.24 ± 0.02 ^f^

All data are expressed as mean ± standard deviation (SD) of triplicate experiments (*n* = 3). Different superscript letters indicate significantly different total phenolic contents and antioxidant activities of different bean cultivars in the same assay (*p* < 0.05) using one-way analysis of variance (ANOVA) and Duncan’s multiple comparison test. GAE: gallic acid equivalent; TE: Trolox equivalent; DW: dry weight; ORAC: oxygen radical absorbance capacity; FRAP: ferric ion reducing antioxidant power; DPPH: 2,2-diphenyl-1-picrylhydrazyl.

## Data Availability

Data are contained within this article and Supplementary Material.

## Supplementary Materials

The following supporting information can be downloaded at: https://www.mdpi.com/article/10.3390/foods11142062/s1, Supplementary Table S1. Morphological characteristics (seed width, length, and thickness) of ten bean cultivars; Supplementary Table S2. Color values of ten bean cultivars (fresh seeds and powder samples); Supplementary Table S3. Proximate compositions of ten bean cultivars (per 100 g fresh weight); Supplementary Table S4. Vitamin contents of ten bean cultivars (per 100 g fresh weight); Supplementary Table S5. Mineral contents of ten bean cultivars (per 100 g fresh weight); Supplementary Table S6. Proximate compositions of *Glycine max* (L.) Merrill cultivars ‘SJ5’ and ‘CM60’ compared with data from the USDA FDC and the Thai FCD databases (per 100 g dry weight); Supplementary Table S7. Proximate compositions of *Phaseolus lunatus* L cultivars ‘38’, ‘47’, and ‘59’ compared with data from the USDA FDC and the PulseDM databases (per 100 g dry weight); Supplementary Table S8. Proximate compositions of *Phaseolus vulgaris* L. cultivar ‘112’ compared with data from PulseDM, the USDA FDC, and Thai FCD databases (per 100 dry weight); Supplementary Table S9. Proximate compositions of *Vigna umbellata* (Thunb.) Ohwi and H.Ohashi cultivar ‘107’ and *Vigna angularis* (Wild.) Ohwi and Ohashi cultivar ‘108’ compared with data from PulseDM and the USDA FDC databases (per 100 g dry weight); Supplementary Table S10. Proximate compositions of *Vigna mungo* (L.) Hepper cultivar ‘CN4’ and *Vigna radiata* (L.) Wilczek cultivar ‘CN84-1’ compared with data from PulseDM, the USDA FDC, and Thai FCD databases (per 100 g dry weight).
