# Supplementary material for: Nutritional Compositions, Phenolic Contents, and Antioxidant Potentials of Ten Original Lineage Beans in Thailand
Source: Foods. 2022 Jul 12;11(14):2062. doi: 10.3390/foods11142062 (PMC9324593; doi:10.3390/foods11142062)
Supplement: Supplementary file 1 [file foods-11-02062-s001.zip › foods-1777287-supplementary.pdf]

foods

Supplementary materials

Nutritional Compositions, Phenolic Contents, and Antioxidant Potentials of Ten Original Lineage Beans in Thailand

Yuraporn Sahasakul [1], Amornrat Aursalung [1], Sirinapa Thangsiri [1], Pitthaya Wongchang [2], Parichart Sangkasa-ad [2], Aphinya Wongpia [2], Auytin Polpanit [3], Woorawee Inthachat [1] , Piya Temviriyanukul [1] and Uthaiwan Suttisansanee [1,*]

[1] Food and Nutrition Academic and Research Cluster, Institute of Nutrition, Mahidol University, Salaya, Phuttamonthon, Nakhon Pathom 73170, Thailand; yuraporn.sah@mahidol.ac.th (Y.S.); amornrat.aur@mahidol.ac.th (A.A.); sirinapa.tha@mahidol.ac.th (S.T.); woorawee.int@mahidol.ac.th (W.I.); piya.tem@mahidol.ac.th (P.T.)

[2] Biotechnology Research and Development Office, Department of Agriculture Rangsit-Nakorn Nayok, Rangsit (Klong 6), Thanyaburi, Pathum Thani 12100, Thailand; pitthaya@hotmail.com (P.W.); psk50_2003@hotmail.com (P.S.-a.); aphinya.wongpia@gmail.com (A.W.)

[3] Chiang Mai Field Crops Research Center, Department of Agriculture, Nong Han, San Sai District, Chiang Mai 50290, Thailand; auytin1804@hotmail.com (A.P.)

* Correspondence: uthaiwan.sut@mahidol.ac.th (U.S.); Tel.: +66-(0)-2800-2380 (ext.422)

Supplementary Table S1:

Morphological characteristics (seed width, length, and thickness) of ten bean cultivars.

Cultivars	Seed width (mm)	Seed length (mm)	Seed thickness (mm)
38	10.04 ± 0.72	14.54 ± 0.87	4.71 ± 1.93
47	8.56 ± 0.62	12.89 ± 0.66	4.55 ± 1.29
59	9.87 ± 0.80	14.55 ± 1.39	4.80 ± 1.64
112	6.25 ± 0.77	16.10 ± 1.18	6.19 ± 0.51
107	4.60 ± 0.44	7.61 ± 0.53	3.65 ± 0.27
108	4.93 ± 0.36	7.24 ± 0.58	4.94 ± 0.50
CN4	3.84 ± 0.39	4.87 ± 0.47	3.56 ± 0.33
CN84-1	4.03 ± 0.26	5.65 ± 0.62	3.93 ± 0.09
SJ5	6.00 ± 0.47	7.27 ± 0.47	4.83 ± 0.46
CM60	6.53 ± 0.26	7.39 ± 0.32	5.89 ± 0.47

All data were expressed as mean ± standard deviation (SD) of triplicate experiments (n = 3). Seed sizes were determined using a 0.01mm/0.0005" digital Vernier.

Supplementary Table S2:

Color values of ten bean cultivars (fresh seeds and powdered samples).

Cultivars	Color values					
	Fresh Seeds			Powdered Samples		
	L*	a*	b*	L*	a*	b*
38	36.80 ± 1.34	11.87 ± 0.32	14.69 ± 0.09	54.43 ± 0.33	0.78 ± 0.10	7.37 ± 0.34
47	77.84 ± 0.14	2.15 ± 0.04	16.42 ± 0.06	58.59 ± 0.46	0.18 ± 0.05	10.26 ± 0.41
59	17.33 ± 0.33	4.42 ± 0.38	0.94 ± 0.05	54.23 ± 4.08	0.42 ± 0.02	6.51 ± 0.23
112	25.25 ± 1.22	15.89 ± 0.85	8.00 ± 0.72	54.26 ± 0.11	1.62 ± 0.01	4.98 ± 0.07
107	28.62 ± 0.25	10.45 ± 0.13	9.23 ± 0.16	52.33 ± 0.37	0.54 ± 0.04	5.88 ± 0.06
108	23.69 ± 0.29	14.76 ± 0.23	8.37 ± 0.03	51.82 ± 0.01	1.38 ± 0.01	6.10 ± 0.04
CN4	26.88 ± 0.10	1.25 ± 0.03	6.35 ± 0.08	52.86 ± 0.24	(-0.01) ± 0.02	5.44 ± 0.07
CN84-1	35.02 ± 0.08	0.85 ± 0.02	26.24 ± 0.07	53.84 ± 0.61	(-0.10) ± 0.05	10.17 ± 0.10
SJ5	59.24 ± 0.06	9.10 ± 0.03	31.55 ± 0.03	53.75 ± 0.36	1.80 ± 0.12	18.61 ± 0.45
CM60	57.46 ± 0.03	9.12 ± 0.01	32.41 ± 0.02	54.33 ± 0.24	1.16 ± 0.29	17.61 ± 0.22

All data were expressed as mean ± standard deviation (SD) of triplicate experiments ($n = 3$). The colors were analyzed using a ColorFlex EZ spectrophotometer and expressed as CIELAB units, in which L* represents darkness (0) to lightness (100), a* represents green (-) to red (+) colors, and b* represents blue (-) to yellow (+) colors.

Supplementary Table S3:

Proximate compositions of ten bean cultivars (per 100 g fresh weight).

Cultivars	Energy (kcal)	Moisture (g)	Protein (g)	Fat (g)	Carbohydrate (g)	Dietary fiber (g)	Ash (g)
38	354.57 ± 1.37 ^{e}	9.62 ± 0.23 ^{b}	20.31 ± 0.11 ^{g}	1.15 ± 0.11 ^{de}	65.76 ± 0.00 ^{c}	19.29 ± 0.40 ^{c}	3.18 ± 0.01 ^{i}
47	356.02 ± 0.19 ^{e}	9.06 ± 0.28 ^{c}	15.12 ± 0.19 ^{i}	1.56 ± 0.25 ^{c}	70.38 ± 0.70 ^{a}	19.94 ± 0.21 ^{b}	3.89 ± 0.01 ^{e}
59	349.35 ± 0.92 ^{f}	10.37 ± 0.33 ^{a}	17.21 ± 0.30 ^{h}	1.19 ± 0.04 ^{de}	67.45 ± 0.02 ^{b}	17.22 ± 0.31 ^{e}	3.79 ± 0.04 ^{f}
112	361.70 ± 0.95 ^{c}	6.39 ± 0.14 ^{e}	21.53 ± 0.06 ^{f}	1.30 ± 0.24 ^{cd}	65.98 ± 0.23 ^{c}	19.59 ± 0.18 ^{bc}	4.81 ± 0.09 ^{c}
107	350.25 ± 1.24 ^{f}	9.44 ± 0.35 ^{bc}	20.46 ± 0.02 ^{g}	0.95 ± 0.02 ^{ef}	64.98 ± 0.32 ^{d}	18.07 ± 0.08 ^{d}	4.18 ± 0.02 ^{d}
108	360.94 ± 1.29 ^{cd}	6.51 ± 0.19 ^{e}	25.20 ± 0.05 ^{c}	0.72 ± 0.11 ^{f}	63.42 ± 0.13 ^{e}	16.59 ± 0.32 ^{f}	4.16 ± 0.00 ^{d}
CN4	359.84 ± 0.32 ^{d}	7.96 ± 0.16 ^{d}	23.40 ± 0.16 ^{e}	1.02 ± 0.11 ^{def}	64.28 ± 0.48 ^{d}	21.39 ± 0.03 ^{a}	3.35 ± 0.05 ^{h}
CN84-1	371.35 ± 0.67 ^{b}	5.12 ± 0.10 ^{f}	24.96 ± 0.08 ^{d}	1.33 ± 0.05 ^{cd}	64.89 ± 0.02 ^{d}	11.92 ± 0.12 ^{h}	3.71 ± 0.01 ^{g}
SJ5	458.72 ± 0.78 ^{a}	5.48 ± 0.10 ^{f}	38.19 ± 0.08 ^{a}	20.06 ± 0.12 ^{b}	31.36 ± 0.16 ^{g}	15.69 ± 0.03 ^{g}	4.92 ± 0.05 ^{b}
CM60	457.62 ± 0.36 ^{a}	6.57 ± 0.39 ^{e}	34.28 ± 0.08 ^{b}	20.98 ± 0.36 ^{a}	32.93 ± 0.80 ^{f}	15.94 ± 0.10 ^{g}	5.26 ± 0.02 ^{a}

All data were expressed as mean ± standard deviation (SD) of triplicate experiments (n = 3). Different superscript letters indicate significantly different contents of the same proximate composition in different bean cultivars ($p < 0.05$) using one-way analysis of variance (ANOVA) and Duncan's multiple comparison test.

Supplementary Table S4:

Vitamin contents of ten bean cultivars (per 100 g fresh weight).

Cultivars	Vitamin B1 (mg)	Vitamin B2 (mg)	Vitamin B3 (mg)
38	0.47 ± 0.02 ^{a}	0.03 ± 0.00 ^{c}	1.19 ± 0.01 ^{d}
47	0.34 ± 0.01 ^{c}	0.03 ± 0.01 ^{c}	1.30 ± 0.01 ^{bc}
59	0.32 ± 0.01 ^{c}	0.03 ± 0.00 ^{c}	1.21 ± 0.01 ^{cd}
112	0.47 ± 0.00 ^{a}	0.03 ± 0.01 ^{c}	0.48 ± 0.06 ^{f}
107	0.13 ± 0.02 ^{e}	0.03 ± 0.00 ^{c}	1.41 ± 0.04 ^{a}
108	0.04 ± 0.00 ^{g}	0.02 ± 0.00 ^{d}	0.90 ± 0.14 ^{e}
CN4	0.41 ± 0.02 ^{b}	0.10 ± 0.00 ^{a}	1.28 ± 0.04 ^{bcd}
CN84-1	0.16 ± 0.02 ^{d}	0.04 ± 0.00 ^{b}	1.29 ± 0.01 ^{bc}
SJ5	0.43 ± 0.02 ^{b}	0.03 ± 0.00 ^{c}	1.35 ± 0.02 ^{ab}
CM60	0.07 ± 0.00 ^{f}	0.03 ± 0.00 ^{c}	1.22 ± 0.02 ^{cd}

All data were expressed as mean ± standard deviation (SD) of triplicate experiments (n = 3). Different superscript letters indicate significantly different contents of the same vitamin in different bean cultivars ($p < 0.05$) using one-way analysis of variance (ANOVA) and Duncan's multiple comparison test.

Supplementary Table S5:

Mineral contents of ten bean cultivars (per 100 g fresh weight).

Cultivars	Macromineral (mg)				Micromineral (mg)	
	Calcium	Sodium	Potassium	Magnesium	Iron	Zinc
38	78.09 ± 1.23 ^{f}	30.15 ± 7.80 ^{c}	894.99 ± 38.09 ^{ef}	104.36 ± 0.14 ^{i}	3.89 ± 0.39 ^{ef}	1.74 ± 0.03 ^{g}
47	86.28 ± 0.39 ^{e}	25.79 ± 0.46 ^{cd}	1215.55 ± 32.15 ^{c}	126.20 ± 1.82 ^{e}	4.33 ± 0.58 ^{e}	1.87 ± 0.05 ^{f}
59	90.06 ± 0.78 ^{de}	17.56 ± 1.86 ^{de}	1232.86 ± 19.82 ^{c}	115.55 ± 1.83 ^{f}	3.70 ± 0.13 ^{f}	1.82 ± 0.01 ^{fg}
112	97.47 ± 1.81 ^{c}	50.05 ± 9.24 ^{b}	1420.42 ± 18.67 ^{a}	111.35 ± 0.38 ^{g}	6.55 ± 0.07 ^{b}	2.23 ± 0.01 ^{e}
107	28.95 ± 0.33 ^{h}	15.17 ± 1.21 ^{e}	1041.71 ± 7.41 ^{d}	158.33 ± 0.88 ^{c}	4.88 ± 0.27 ^{d}	2.63 ± 0.02 ^{c}
108	66.87 ± 1.07 ^{g}	69.15 ± 7.85 ^{a}	1355.18 ± 26.22 ^{b}	107.32 ± 0.45 ^{h}	7.07 ± 0.32 ^{a}	2.18 ± 0.01 ^{e}
CN4	107.83 ± 1.36 ^{b}	42.33 ± 4.03 ^{b}	842.45 ± 27.63 ^{f}	156.65 ± 0.26 ^{c}	5.56 ± 0.22 ^{c}	2.72 ± 0.01 ^{c}
CN84-1	91.90 ± 2.45 ^{d}	20.44 ± 0.64 ^{cde}	922.76 ± 5.12 ^{e}	132.63 ± 0.41 ^{d}	3.77 ± 0.12 ^{f}	2.42 ± 0.02 ^{d}
SJ5	228.17 ± 0.94 ^{a}	19.50 ± 1.62 ^{de}	1181.13 ± 4.25 ^{c}	164.22 ± 1.03 ^{b}	6.53 ± 0.25 ^{b}	4.17 ± 0.07 ^{a}
CM60	226.25 ± 6.67 ^{a}	44.51 ± 8.35 ^{b}	1390.06 ± 73.49 ^{ab}	210.04 ± 2.56 ^{a}	6.20 ± 0.23 ^{b}	4.03 ± 0.16 ^{b}

All data were expressed as mean ± standard deviation (SD) of triplicate experiments (n = 3). Different superscript letters indicate significantly different contents of the same mineral in different bean cultivars ($p < 0.05$) using one-way analysis of variance (ANOVA) and Duncan's multiple comparison test.

Supplementary Table S6:

Proximate compositions of *Glycine max* (L.) Merrill cultivars 'SJ5' and 'CM60' compared with data from the USDA FDC and the Thai FCD databases (per 100 g dry weight).

Samples	Soybean 'SJ5'	Soybean 'CM60'	Soybean (USDA FDC [a])	Soybean (Thai FCD [b])
Energy (kcal)	485.32	489.78	488	477 (382) [c]
Protein (g)	40.40	36.38	39.91	39.79
Fat (g)	21.22	22.45	21.76	19.92
Carbohydrate (g)	33.17	35.24	33.02	34.92 (10.83) [d]
Dietary fiber (g)	16.59	17.06	10.17	24.09
Ash (g)	5.20	5.62	5.32	5.80
Vitamin B1 (mg)	0.45	0.07	0.96	1.27
Vitamin B2 (mg)	0.03	0.03	0.95	0.32
Vitamin B3 (mg)	1.43	1.30	1.77	2.85
Calcium (mg)	241.39	242.17	302.86	279.43
Sodium (mg)	20.63	47.61	2.19	18.70
Potassium (mg)	1249.61	1487.96	1968.07	1970.30
Magnesium (mg)	173.74	224.81	306.14	NA
Iron (mg)	6.91	6.63	17.17	9.69
Zinc (mg)	4.41	4.31	5.35	3.21

[a] U.S. Department of Agriculture (USDA), Food Data Central (FDC) Database: soybean (FDC ID 174270), calculated to dry weight basis using moisture content of 8.54%.

[b] Thai Food Composition Database: soybean (Food code C33), calculated to dry weight basis using moisture content of 9.1%.

[c] (Energy) was calculated based on the following equation; energy = (protein × 4) + (fat × 9) + (available carbohydrate × 4) + (dietary fiber × 2).

[d] (Carbohydrate) demonstrated available carbohydrate.

NA = data not available

Supplementary Table S7:

Proximate compositions of *Phaseolus lunatus* L cultivar ′38′, ′47′ and ′59′ compared with data from the USDA FDC and the PulseDM databases (per 100 g dry weight).

Samples	Lima bean '38′	Lima bean '47′	Lima bean '59′	Lima bean (USDA FDC [a])	Lima bean (PulseDM [b])
Energy (kcal)	392.28	391.47	389.75	385	390 (348) [c]
Protein (g)	22.47	16.62	19.20	23.9	23.0
Fat (g)	1.27	1.72	1.33	0.77	1.7
Carbohydrate (g)	72.75	77.39	75.25	70.60	70.6 (49.6) [d]
Dietary fiber (g)	21.34	21.93	19.21	21.20	21.0
Ash (g)	3.51	4.28	4.22	4.80	4.6
Vitamin B1 (mg)	0.51	0.37	0.36	0.56	0.6
Vitamin B2 (mg)	0.03	0.03	0.03	0.22	0.2
Vitamin B3 (mg)	1.31	1.42	1.34	1.71	1.8
Calcium (mg)	86.40	94.87	100.47	90.2	74.0
Sodium (mg)	33.34	28.36	19.59	20.10	8.0
Potassium (mg)	990.13	1336.65	1375.48	1915.4	1670.0
Magnesium (mg)	115.46	138.76	128.91	249.44	194.0
Iron (mg)	4.30	4.75	4.12	8.36	7.2
Zinc (mg)	1.93	2.05	2.03	3.15	3.05

[a] U.S. Department of Agriculture (USDA), Food Data Central (FDC) Database: lima bean (FDC ID 174252), calculated to dry weight basis using moisture content of 10.2%.
[b] FAO/INFOODS Global database for pulses on a dry matter basis (PulseDM): lima bean (Food ID PHL001_DM).
[c] (Energy) was calculated based on the following equation; energy = (protein × 4) + (fat × 9) + (available carbohydrate × 4) + (dietary fiber × 2).
[d] (Carbohydrate) demonstrated available carbohydrate.

Supplementary Table S8:

Proximate compositions of *Phaseolus vulgaris* L.cultivar '112' compared with data from the PulseDM, the USDA FDC, and the Thai FCD databases (per 100 dry weight).

Samples	Red kidney bean '112'	Red kidney bean (PulseDM [a])	Red kidney bean (USDA FDC [b])	Red kidney bean (Thai FCD [c])
Energy (kcal)	386.39	392 (344) [d]	391	392 (335) [d]
Protein (g)	23.00	25.6	25.51	23.04
Fat (g)	1.38	1.8	1.20	2.12
Carbohydrate (g)	70.48	68.4 (44.2) [e]	69.50	70.30 (41.6) [e]
Dietary fiber (g)	20.93	24.2	17.23	28.73
Ash (g)	5.13	4.0	3.82	4.59
Vitamin B1 (mg)	0.50	0.52	0.69	NA
Vitamin B2 (mg)	0.03	0.2	0.24	1.49
Vitamin B3 (mg)	0.51	2.7	2.39	2.99
Calcium (mg)	104.12	98	94.10	107.47
Sodium (mg)	53.48	10	13.61	NA
Potassium (mg)	1517.36	1460	1541.95	NA
Magnesium (mg)	118.95	167	156.46	NA
Iron (mg)	7.00	9.9	7.59	7.76
Zinc (mg)	2.38	3.63	3.16	NA

[a] FAO/INFOODS Global database for pulses on a dry matter basis (PulseDM): red kidney bean (Food ID PHV004_DM).
[b] U.S. Department of Agriculture (USDA), Food Data Central (FDC) Database: red kidney bean (FDC ID 173744), calculated to dry weight basis using moisture content of 11.8%.
[c] Thai Food Composition Database (Thai FCD): red kidney bean (Food code C18), calculated to dry weight basis using moisture content of 11.6%.
[d] (Energy) was calculated based on the following equation; energy = (protein × 4) + (fat × 9) + (available carbohydrate × 4) + (dietary fiber × 2).
[e] (Carbohydrate) demonstrated available carbohydrate.
NA = data not available

Supplementary Table S9:

Proximate compositions of *Vigna umbellata* (Thunb.) Ohwi & H.Ohashi cultivar ′107′ and *Vigna angularis* (Wild.) Ohwi & Ohashi ′108′ compared with data from the PulseDM and the USDA FDC databases (per 100 g dry weight).

Samples	Red rice bean '107′	Red rice bean (Thai FCD [a])	Rice bean (PulseDM [b])	Azuki bean '108′	Azuki bean (PulseDM [b])	Azuki bean (USDA FDC [c])
Energy (kcal)	386.76	396 (337) [d]	385 (355) [d]	386.07	387 (357) [d]	388
Protein (g)	22.59	25.51	21.2	26.95	23	22.98
Fat (g)	1.04	2.45	0.6	0.77	0.6	0.61
Carbohydrate (g)	71.75	67.95 (38.33) [e]	73.6 (58.6) [e]	67.84	72.3 (57.6) [e]	72.63
Dietary fiber (g)	19.95	29.62	15.0	17.75	14.7	14.67
Ash (g)	4.62	4.11	4.5	4.44	4.1	3.76
Vitamin B1 (mg)	0.14	1.11	0.52	0.04	0.53	0.53
Vitamin B2 (mg)	0.03	0.31	0.29	0.02	0.25	0.25
Vitamin B3 (mg)	1.55	2.49	3.7	0.96	2.9	3.04
Calcium (mg)	31.97	77.95	322	71.53	84	76.21
Sodium (mg)	16.75	17.82	41	73.97	6	5.77
Potassium (mg)	1150.33	1316.26	1570	1449.51	1390	1443.42
Magnesium (mg)	174.83	NA	227	114.79	144	5.75
Iron (mg)	5.39	7.33	7	7.56	5.2	5.75
Zinc (mg)	2.90	2.67	3.2	2.33	5.64	5.82

[a] Thai Food Composition Database (Thai FCD): red rice bean (Food code C16), calculated to dry weight basis using moisture content of 10.2%.

[b] FAO/INFOODS Global database for pulses on a dry matter basis (PulseDM): rice bean (Food ID VIU002_DM), azuki bean (Food ID VIA001_DM).

[c] U.S. Department of Agriculture (USDA), Food Data Central (FDC) Database: azuki bean (FDC ID 173727), calculated to dry weight basis using moisture content of 13.4%.

[d] (Energy) was calculated based on the following equation; energy = (protein ×x 4) + (fat × 9) + (available carbohydrate × 4) + (dietary fiber × 2).

[e] (Carbohydrate) demonstrated available carbohydrate.

NA = data not available

Supplementary Table S10:

Proximate compositions of *Vigna mungo* (L.) Hepper cultivar ′CN4′ and *Vigna radiata* (L.) Wilczek cultivar ′CN84-1′ compared with data from the PulseDM, the USDA FDC, and the Thai FCD databases (per 100 g dry weight).

Samples	Black gram 'CN4'	Mung bean 'CN84-1'	Mungo bean (PulseDM [a])	Mungo bean (USDA FDC [b])	Mung bean (PulseDM [a])	Mung bean (USDA FDC [b])	Mung bean (Thai FCD [c])
Energy (kcal)	390.96	391.37	392 (350) [d]	394.13	394 (360) [d]	391.81	394 (335) [d]
Protein (g)	25.42	26.3	26.4	28.25	23.2	26.28	25.56
Fat (g)	1.10	1.4	1.5	1.84	1.5	1.26	1.77
Carbohydrate (g)	69.83	68.39	68.3 (46.7) [e]	66.14	71.9 (54.9) [e]	68.83	68.89 (39.86) [e]
Dietary fiber (g)	23.23	12.56	21.6	20.52	17.0	17.92	29.03
Ash (g)	3.64	3.91	3.7	3.77	3.4	3.65	3.79
Vitamin B1 (mg)	0.44	0.16	0.57	0.31	0.41	0.68	0.88
Vitamin B2 (mg)	0.11	0.04	0.26	0.28	0.29	0.26	0.26
Vitamin B3 (mg)	1.39	1.35	2.1	1.63	2.4	2.47	2.74
Calcium (mg)	117.15	96.85	120	154.71	99	145.13	96.77
Sodium (mg)	45.98	21.54	23	42.60	10	16.49	75.64
Potassium (mg)	915.34	972.51	1280	1102.02	1300	1374.38	1231.37
Magnesium (mg)	170.20	139.78	224	299.33	154	207.81	120.13
Iron (mg)	6.04	3.97	8.2	8.49	4.9	7.41	7.79
Zinc (mg)	2.95	2.55	3.37	3.76	1.8	2.95	3.24

[a] FAO/INFOODS Global database for pulses on a dry matter basis (PulseDM): mungo bean (Food ID VIM001_DM) and mung bean (Food ID VIR001_DM).
[b] U.S. Department of Agriculture (USDA), Food Data Central (FDC) Database: mungo bean (FDC ID 174259), calculated to dry weight basis using moisture content of 10.2%, and mung bean (FDC ID 174256), calculated to dry weight basis using moisture content of 9.05%.
[c] Thai Food Composition Database (Thai FCD): mung bean (Food code C15), calculated to dry weight basis using moisture content of 10.1%.
[d] (Energy) was calculated based on the following equation; energy = (protein × 4) + (fat × 9) + (available carbohydrate × 4) + (dietary fiber × 2).
[e] (Carbohydrate) demonstrated available carbohydrate.
